# Relationship between β-Cell Autoantibodies and Their Combination with Anthropometric and Metabolic Components and Microvascular Complications in Latent Autoimmune Diabetes in Adults

**DOI:** 10.3390/biomedicines11092561

**Published:** 2023-09-18

**Authors:** Tomislav Bulum, Marijana Vučić Lovrenčić, Jadranka Knežević Ćuća, Martina Tomić, Sandra Vučković-Rebrina, Lea Duvnjak

**Affiliations:** 1 Department of Diabetes and Endocrinology, Vuk Vrhovac University Clinic for Diabetes, Endocrinology and Metabolic Diseases, Merkur University Hospital, 10000 Zagreb, Croatia; 2 Medical School, University of Zagreb, 10000 Zagreb, Croatia; 3 Clinical Department of Medical Biochemistry and Laboratory Medicine, Merkur University Hospital, 10000 Zagreb, Croatia; 4 Scientific Research Unit, Merkur University Hospital, 10000 Zagreb, Croatia; 5 Department of Ophthalmology, Vuk Vrhovac University Clinic for Diabetes, Endocrinology and Metabolic Diseases, Merkur University Hospital, 10000 Zagreb, Croatia; 6 Department of Neurology, Vuk Vrhovac University Clinic for Diabetes, Endocrinology and Metabolic Diseases, Merkur University Hospital, 10000 Zagreb, Croatia

**Keywords:** autoimmune diabetes in adults, autoimmunity, glutamic acid decarboxylase autoantibodies

## Abstract

Aims: Our study aimed to investigate the relationship between three autoantibodies and their combination with anthropometric and metabolic components and microvascular complications in patients with latent autoimmune diabetes in adults (LADA). Methods: Our study included 189 LADA patients divided into four subgroups according to the autoantibodies present: glutamic acid decarboxylase autoantibodies (GADA) only; zinc transporter-8 autoantibodies (ZnT8A)+GADA; insulinoma-associated-2 autoantibodies (IA-2)+GADA; and ZnT8+IA-2+GADA. Results: Compared to GADA positivity only, patients with ZnT8+GADA positivity and ZnT8+IA-2+GADA positivity had a shorter diabetes duration and lower body mass index (BMI); patients with ZnT8+GADA positivity were younger and showed an increase in glomerular filtration rate, while those with ZnT8+IA-2+GADA positivity had lower C-peptide and lower insulin resistance measured with HOMA2-IR. In a multiple regression analysis, ZnT8 positivity was associated with lower BMI (*p* = 0.0024), female sex (*p* = 0.0005), and shorter duration of disease (*p* = 0.0034), while IA-2 positivity was associated with lower C-peptide levels (*p* = 0.0034) and shorter diabetes duration (*p* = 0.02). No association between antibody positivity and microvascular complications of diabetes, including retinopathy, neuropathy, and microalbuminuria, as well as with variables of glucose control and β-cell function were found. Conclusion: The results of our study suggest that ZnT8 and IA-2 autoantibodies are present in a significant number of LADA patients and associated with clinical and metabolic characteristics resembling classic type 1 diabetes. Due to increased LADA prevalence, earlier identification of patients requiring frequent monitoring with the earlier intensification of insulin therapy might be of special clinical interest.

## 1. Introduction

Type 1 diabetes mellitus (T1DM) and latent autoimmune diabetes in adults (LADA) are autoimmune conditions associated with circulating autoantibodies which indicate the destruction of pancreatic islet β-cells, leading to insulin deficiency and hyperglycemia [1]. Compared to classic type 1 diabetes, which develops in childhood and progresses rapidly, LADA is characterized by a slow progression of the disease and slower pancreatic β-cell dysfunction and usually manifests itself in later adulthood [2,3]. Such patients were previously diagnosed with type 2 diabetes, although most did not have the clinical features of metabolic syndrome. Due to the slow progression of the disease, patients with LADA can achieve optimal glucose control with oral antihyperglycemic agents only for years with preserved β-cell function measured with C-peptide [4]. Diagnosis of LADA is confirmed with a positive test for circulating specific β-cell autoantibodies (glutamic acid decarboxylase enzyme autoantibodies (GADA), zinc transporter-8 autoantibodies (ZnT8), and tyrosine phosphatase-like transmembrane glycoprotein autoantibodies (IA2)) [5,6,7,8]. GADA autoantibodies are predominant in the definition of LADA, although detectable GADA does not always confirm autoimmune diabetes etiology [9]. After verifying the diagnosis of LADA, the main goal of treatment, as in patients with type 1 diabetes and type 2 diabetes, is to achieve optimal glucoregulation to prevent the development and progression of micro- and macrovascular complications.

It is suggested that LADA is not a unique subtype of diabetes but instead represents a combination of two heterogeneous populations with very different phenotypes. One phenotype represents type 1 diabetes and the other represents type 2 diabetes [10]. Single autoantibody positivity and lower titer are more closely related to the type 2 diabetes phenotype, whereas the high titer of GADA as well as simultaneous positivity for ZnT8 and IA2 autoantibodies are more closely associated with the type 1 diabetes phenotype [9,11]. Generally, metabolic syndrome and its component disorders (obesity, blood pressure, and triglycerides) and cardiovascular disease are more common in patients with LADA than in classic type 1 diabetes but less common than in those with classic type 2 diabetes [12,13]. Also, the GADA titer is negatively associated with the age of onset, total cholesterol and triglycerides, obesity, and C-peptide concentrations and positively associated with the hemoglobin A_1_c and high-density lipoprotein cholesterol (HDL-C) concentrations [14]. GADA titer negatively correlates with the C-peptide concentration, and LADA patients with higher GADA titers show more insulin deficiency [15,16]. Different autoantibody titers and their positivity affect LADA patients’ different phenotypes and clinical characteristics [17]. In addition, up to 10% of all patients with type 2 diabetes meet the criteria for LADA [18].

Diabetes-related comorbidities of patients with LADA and positivity for single or simultaneous autoantibodies are not fully understood. Some patients develop micro- and macrovascular complications despite improved glycemic control and independently of metabolic syndrome features [15,19]. Our study aimed to investigate the relationship between three specific β-cell autoantibodies, GADA, IA-2, and ZnT8, and their combination with anthropometric and metabolic components and with microvascular complications in adult patients with autoimmune diabetes.

## 2. Methods

### 2.1. Study Design and Ethics Statement

This cross-sectional study was performed at the Department of Diabetes and Endocrinology and Clinical Department of Medical Biochemistry and Laboratory Medicine at Merkur University Hospital in Zagreb, Croatia. A total of 189 LADA patients aged over 35 years with a minimum of 1 year since being diagnosed with diabetes and GADA-positive autoantibodies were included in the study. The study was conducted following the Declaration of Helsinki and approved by the hospital’s ethics committee (protocol number 05/01-850, approval date: 16 September 2020). All study participants received written and oral information about the study and signed the written informed consent form.

### 2.2. Demographic Data, Medical Records, and Clinical Characteristics

Demographic data of patients included age, gender, and diabetes duration. Body mass index (BMI) was calculated by dividing weight and height squared (kg/m^2^). Weight and height were measured using a balance-beam scale and a wall-mounted stadiometer. Systolic/diastolic blood pressure (SBP/DBP) were measured with an ambulatory digital sphygmomanometer after a 10-min resting period using a cuff appropriate to the length and circumference of the arm and expressed in mmHg.

### 2.3. Measure of β-Cell Function

Fasting venous blood samples were collected to determine the biochemistry panel, including glucose, C-peptide, and HbA_1_c. HbA_1_c was measured in EDTA-anticoagulated whole blood samples using an automated turbidimetric inhibition immunoassay (HbA_1_c Gen 3, Cobas Integra 400 Plus, Roche Diagnostic, Basel, Switzerland), traceable to the International Federation of Clinical Chemistry and Laboratory Medicine (IFCC) reference system and reported in National Glycohemoglobin Standardization Program (NGSP) units (%). Fasting serum C-peptide concentration was determined using an automated chemiluminescent immunoassay (Advia Centaur XP, Siemens Healthineers, Tarrytown, NY, USA). HOMA2-%B and HOMA2-IR were determined using the HOMA2 Calculator (available at https://www.dtu.ox.ac.uk/homacalculator/download.php, accessed on 14 September 2023; Oxford Centre for Diabetes, Endocrinology and Metabolism, Oxford, UK). We used fasting blood glucose and serum C-peptide levels to calculate the HOMA2 score. The secretory capacity of the pancreatic islet β-cells is expressed using the HOMA2 calculator as HOMA2-%B, where the higher the value, the more insulin the β-cells can secrete to respond to blood glucose levels. Higher HOMA2-%B and HOMA2-IR scores indicate increased insulin sensitivity and insulin resistance, respectively.

### 2.4. Detection of Autoantibodies

GAD and IA-2 antibodies were analyzed using their respective bridge ELISA immunoassays (Euroimmun, Lübeck, Germany) calibrated to international units (IU) with the 1st WHO reference reagent for islet cell antibodies (WHO, 1999, reagent 97/550, National Institute for Biological Standards and Control, Hertfordshire, UK). By definition, the NIBSC 97/550 reference reagent contains 100 IU of anti-GAD or anti-IA2 per ampoule. Human recombinant glutamic acid decarboxylase, isoform GAD65, and human recombinant tyrosine phosphatase (IA2) were used as antigens in the respective immunoassays. The lower detection limits for GAD and anti-IA2 are 0.59 IU/mL and 1.04 IU/mL, respectively, and the cut-off values are set at 10 IU/mL for both assays.

ZnT8 autoantibodies were detected using a bridge ELISA (RSR Limited, Cardiff, United Kingdom) capable of detecting and quantifying autoantibodies specific to R 325 (Arg) to W 325 (Trp) or residue 325 non-specific variants. In this assay, ZnT8 Ab in patients’ sera, calibrators, and controls react with ZnT8 coated onto ELISA plate wells. After a 16–20-h incubation, the samples are discarded, ZnT8-Biotin conjugate is added, and ZnT8 Ab in the samples forms a bridge between ZnT8 bound to the wells and ZnT8-Biotin via polyvalent binding. Unbound ZnT8-Biotin is removed by washing, and the amount of bound ZnT8-Biotin is determined by adding streptavidin peroxidase (S-P), which binds specifically to biotin. After washing the unbound S-P, the peroxidase substrate 3,3′, 5,5′ tetramethyl-benzidine (TMB) is added, which results in a chromogenic reaction. After stopping the reaction with acidification, the absorbance of the reaction mixture is read (405/450 nm) using an ELISA plate reader. The absorbance is proportional to the ZnT8 antibody positivity in the sample.

The assay is calibrated against proprietary calibrators manufactured from rabbit serum positive for ZnT8 Ab and values assigned to the RSR arbitrary units. The assay range is 10–2000 U/mL, with a lower detection limit of 1.2 U/mL and the recommended cut-off for positivity at 15 U/mL.

The cut-off limit was 5 IU/mL for GADA, 10 IU/mL for IA2 autoantibodies, and 10 U/mL for ZnT8 autoantibodies, respectively [20]. The patients with autoantibody titers over the established cut-off were considered seropositive [21].

### 2.5. Chronic Complications of Diabetes

Renal function was determined using serum creatinine, glomerular filtration rate (GFR), and albumin/creatinine (A/C) ratio. Serum creatinine was determined using a compensated Jaffe spectrophotometric method traceable to Standard Reference Material 967 provided by the US National Institute of Standards and Technology (NIST). GFR was estimated using the Chronic Kidney Disease Epidemiology Collaboration (CKD-EPI) formula [22], and a random urine sample was collected to determine the A/C ratio using a turbidimetric immunoassay and photometric assay, respectively. Normoalbuminuria was defined as an A/C ratio < 3 mg/mmol and microalbuminuria as an A/C ratio ≥ 3 < 30 mg/mmol. Those with chronic kidney disease, defined as macroalbuminuria (A/C ratio ≥ 30 mg/mmol) and/or estimated GFR < 60 mL/min^−1^1.73 m^−2^, were excluded from the study.

Diabetic retinopathy was diagnosed via binocular indirect slit lamp fundoscopy and color fundus photographs of two fields of both eyes were taken with a suitable 45° fundus camera (VISUCAM, Zeiss, Germany) according to the EURODIAB retinal photography methodology [23]. Evaluation of peripheral sensorimotor neuropathy was based on clinical symptoms (neuropathy symptom score), signs (neuropathy disability score), quantitative sensory testing (vibration perception threshold), and electroneuromyography testing.

### 2.6. Statistical Analysis

The normality of distribution was tested using the Shapiro–Wilk test. Data are expressed as mean ± SD for normally distributed variables and median with range for non-normally distributed variables. Pearson’s and Spearman’s rank correlations were used to assess the relationship between normally and non-normally distributed variables, respectively. Depending on the data distribution, differences between the groups were examined using parametric (*t*-test) or non-parametric tests (Mann–Whitney). A *p*-value of less than 0.05 was considered statistically significant in all analyses. Statistical analysis was performed using Stata/IC ver. 14.2, StataCorp LLC, and MedCalc Statistical Software version 18.11.6 (MedCalc Software bvba, Ostend, Belgium; https://www.medcalc.org; accessed on 1 January 2019).

## 3. Results

**Figure 1:**
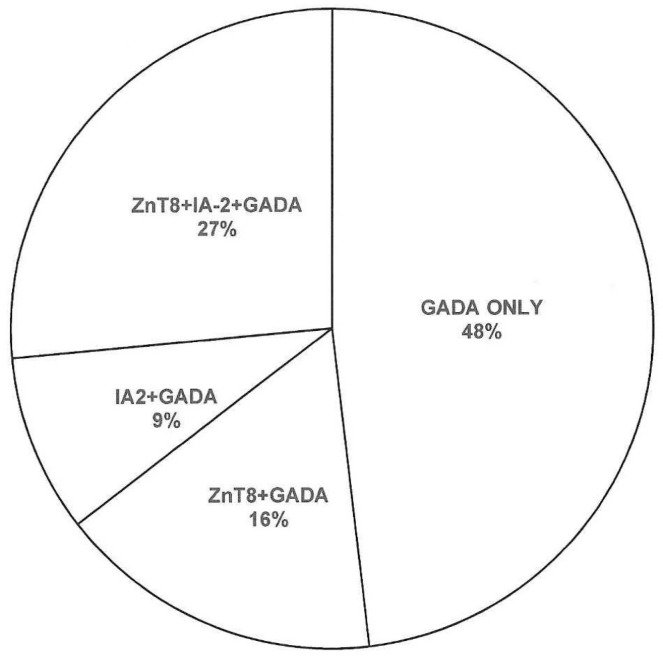
Distribution of β-cell autoantibodies in GADA-positive adult patients with diabetes. GADA: glutamic acid decarboxylase antibodies; ZnT8: zinc transporter-8; IA-2: anti-islet-2 antibodies.

Among 189 adult subjects (91 males (M) and 98 females (F)) with diabetes and GAD-positive autoantibodies included in the study, ZnT8 autoantibodies were detected in 81 subjects (M/F = 38/53) and IA-2 autoantibodies in 67 subjects (M/F = 27/40) while 91 subjects (M/F = 53/38) had only GADA positivity. Regarding autoantibody combinations, 31/17 GADA-positive subjects had ZnT8/IA-2 autoantibody positivity, whereas, in 50 subjects, all three antibodies were detected (Figure 1).

### 3.1. Autoantibodies and Clinical Phenotype

There were significant differences in age, BMI, diabetes duration, C-peptide levels, HOMA2-IR, and eGFR between four subgroups of patients with various antibody patterns (GADA only, ZnT8+GADA; IA-2+GADA; and ZnT8+IA-2+GADA). Patients with a combination of either ZnT8+GADA or ZnT8+IA-2+GADA positivity had a shorter diabetes duration and lower BMI than patients who had GADA only. Compared to the GADA-only positive subgroup, patients with the ZnT8+GADA combination were younger and had a higher eGFR, while those with a combination of all three autoantibodies had lower C-peptide and HOMA2-IR values. Also, various antibody patterns were unevenly distributed according to sex, with predominately males in the subgroups of GADA only and IA-2+GADA positivity, while there were more females in the subgroups of ZnT8-positive patients, either combined with GADA alone or IA-2+GADA (χ^2^ = 10.49, df = 3, *p* = 0.0148).

Males had significantly higher GADA titers than females (*p* = 0.034; Mann–Whitney test), whereas no sex-related differences were observed in other parameters except for BMI in the entire study cohort.

No significant differences in the variables of fasting plasma glucose, HbA_1_c, HOMA2-estimated β-cell function, TSH, and albuminuria between the subgroups of patients with various antibody patterns were observed (Table 1).

**Figure 2:**
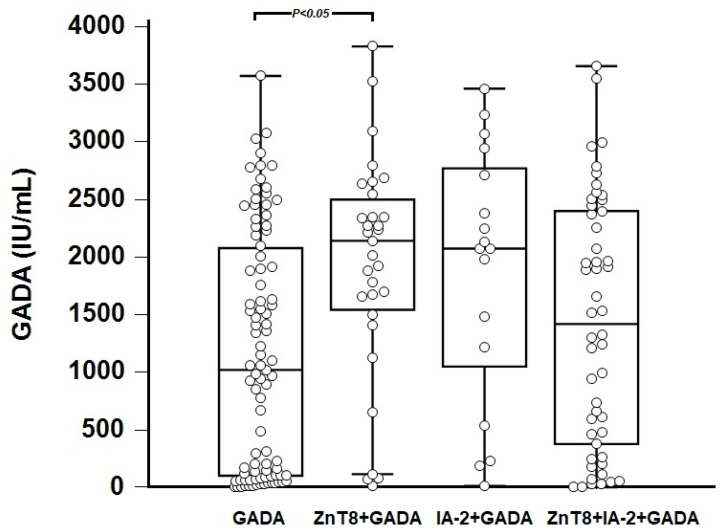
GADA titers in subgroups of patients with various diabetes-specific autoantibody patterns. GADA-only autoantibodies were present in 91 patients, ZnT8+GADA in 31 patients, IA-2+GADA in 17 patients, and ZnT8+IA-2+GADA in 50 patients. The results are medians/IQR (Kruskal–Wallis ANOVA followed by Dunn’s post-hoc test). GADA: glutamic acid decarboxylase antibodies; ZnT8: zinc transporter-8; IA-2: anti-islet-2 antibodies.

GADA titers were significantly higher in the ZnT8+GADA subgroup but not in the IA-2+GADA and ZnT8+IA-2+GADA-positive subgroup when compared to GADA-only positive patients (Figure 2). There were no significant differences in the titers of ZnT8 and IA-2 autoantibodies between the subgroups of ZnT8+GADA and ZnT8+IA-2+GADA-positivity (*p* = 0.1423) and IA-2+GADA and ZnT8+IA-2+GADA-positivity (*p* = 0.0576), respectively (Mann–Whitney U test).

ZnT8 positivity was associated with lower BMI (OR/C.I. = 0.8788/0.8086 to 0.9551, *p* = 0.0024), female sex (OR/C.I. = 4.0371/1.8325 to 8.8960, *p* = 0.0005), and shorter duration of disease (OR/C.I = 0.8933/0.8284 to 0.9633, *p* = 0.0034). On the other hand, IA-2 positivity could be predicted with lower C-peptide levels (OR/C.I. = 0.0675/0.0111 to 0.4102, *p* = 0.0034) and shorter duration of disease (OR/C.I. = 0.9347/0.8829 to 0.9896, *p* = 0.02025). Other variables included in the logistic regression model, including GADA titer, HOMA2-B, HOMA2-IR, eGFR, and HbA_1_c, could not predict ZnT8 and IA-2 antibody positivity, respectively. Thirty-four patients (17%) had hypothyroidism, assessed as either TSH level > 4.5 mIU/L, levothyroxine replacement therapy, or both. GADA titer and HOMA2-B were significant predictors of hypothyroidism (OR/C.I. = 1.0011/1.0006 to 1.0016, *p* = 0.0001 and 1.0228/1.0042 to 1.0417, *p* = 0.0160, respectively). There were no associations between thyroid status and age, sex, duration of diabetes, BMI, and ZnT8/IA-2 positivity in the logistic regression model used.

### 3.2. Associations with Glucose Control and Complications

A multiple logistic regression analysis revealed that GADA titer, duration of diabetes, and HOMA2-estimated β-cell function significantly predicted glucose control, set at an HbA_1_c cut-off of 7.0%, while age, sex, BMI, HOMA2-IR, and positivity for IA-2 and ZnT8 antibodies did not influence glucose control (Table 2). The model was significant (χ^2^ = 37.6, *p* < 0.0001) and correctly classified 71.3% of the cases (AUC = 0.783, SE = 0.038; C.I. = 0.7007 to 0.847).

No association between antibody positivity and microvascular complications of diabetes including retinopathy, neuropathy, and microalbuminuria were found. Also, neither BMI nor β-cell function, insulin resistance, HbA_1_c, sex, and eGFR were significant determinants of microvascular complications in the multiple logistic regression model. However, the duration of diabetes had a remarkable effect, significantly predicting retinopathy, neuropathy, and microalbuminuria (OR/C.I. = 1.1499/1.0713 to 1.2344, *p* = 0.0001; 1.1169/1.0321 to 1.2087, *p* = 0.0060 and 1.0846/1.0257 to 1.1468, *p* = 0.0044, respectively). Furthermore, hypertension was positively associated with microalbuminuria (OR/C.I. = 4.7287/1.8996 to 11.7713, *p* = 0.008), and age was positively associated with neuropathy (OR/C.I. = 1.0639/1.0252 to 1.1041, *p* = 0.0011).

## 4. Discussion

This cross-sectional study included GADA-positive adult diabetic patients classified according to the presence of other β-cell autoimmune markers (ZnT8 and IA2) into groups with single, double, and triple antibody positivity. Our study included only GADA-positive adult diabetic patients because it is questionable whether positivity for a single low-titer islet autoantibody other than GADA is a reliable indicator of autoimmune disease. GADA titers were significantly higher in the ZnT8+GADA subgroup but not in the IA-2+GADA and ZnT8+IA-2+GADA-positive subgroups when compared to GADA-only positive patients. Although ZnT8 is an autoantibody antigen used to diagnose type 1 diabetes, ZnT8 autoantibodies are also detected in many patients with LADA. Unlike GADA and IA-2, the ZnT8 autoantibody is exclusively expressed in pancreatic β-cells and targets the autoimmune process in adult-onset diabetes [24]. In addition, the prevalence of ZnT8 autoantibodies is low in younger individuals but increases dramatically from 3 years onward, peaking at 80% in late adolescence and tending to decline after that [25]. We also observed more females in the subgroups of ZnT8-positive patients combined with GADA alone or IA-2+GADA. It is well known that females have an increased incidence of autoimmune diseases generally, although no gender difference in the incidence of type 1 diabetes was found [26]. ZnT8 autoantibodies were detected in a significant proportion of our patients and thus seem to be a valuable marker of patients with adult-onset autoimmune diabetes. On the contrary, it is suggested that GADA and/or IA-2 antibody positivity in middle-aged individuals at risk for diabetes is not a clinically relevant risk factor for progression to diabetes [27].

Patients with a combination of either ZnT8+GADA or ZnT8+IA-2+GADA positivity had a shorter diabetes duration, lower C-peptide and HOMA2-IR values, and younger age with lower BMI than patients who had GADA only. Compared to only GADA-positive adult diabetic patients, those with multiple antibodies exhibited characteristics more similar to those of type 1 diabetes, with a younger age at disease onset, lower BMI, lower C-peptide level, and higher insulin sensitivity [24,28]. Multiple autoantibody positivity can identify patients with a more severe form of β-cell exhaustion in whom early insulinization might be needed to preserve β-cell function [29]. Those with a single GADA positivity exhibited characteristics similar to those with classic type 2 diabetes. In a clinical setting, it is essential to differentiate these two clinical phenotypes within the adult patient population because the prevalence of LADA has reached a high level in some countries. Nowadays, there is an increased prevalence of LADA in up to 5.9% of newly diagnosed patients with type 2 diabetes over the age of 30 and in almost 65% of patients with newly diagnosed T1DM in China [30].

A multiple logistic regression analysis revealed that GADA titer, along with the duration of diabetes and HOMA2-estimated β-cell function, significantly predicted glucose control, set at an HbA_1_c cut-off of 7.0%, while IA-2 and ZnT8 antibodies did not influence glucose control. Patients with a high GADA titer have an accelerated loss of β-cell function early in the development of the disease, and those with a high GADA titer have worse glycemic control despite higher insulin requirements [31]. In contrast, in patients with type 1 diabetes at diabetes diagnosis, there were no differences in the ZnT8 autoantibody status according to HbA_1_c, and during diabetes follow-up, there were no statistically significant differences in glycemic control and vascular complications related to ZnT8 autoantibody positivity [32]. In addition, in patients with type 2 diabetes, although ZnT8-positive participants experienced a loss of glycemic control during treatment, they exhibited lower rates of diabetic complications than other groups [33].

No association between autoantibody positivity and microvascular complications of diabetes including retinopathy, neuropathy, and microalbuminuria were found in our study. The apparent explanation is that our cohort had a short duration of diabetes (4 years) with reasonably controlled glycemia (median HbA_1_c 7.4%). However, as previously mentioned, ZnT8 antibody positivity in patients with type 2 diabetes was associated with a lower rate of diabetic complications despite a loss of glycemic control on treatment [33]. Also, GAD-positive patients with recent onset of type 1 diabetes reported worse glycemic control but without clinical neuropathy and slightly decreased somatosensory and autonomic nerve function [34]. In our study, patients with single GADA and ZnT8+GADA positivity had a marginally higher (non-significant) risk of microalbuminuria. However, it was documented that in LADA patients, compared with the GADA-negative patients, microalbuminuria was less frequent both at baseline and during follow-up [35]. In another study, patients with GADA had a significantly lower prevalence of diabetic retinopathy compared with patients without GADA (19.2 vs. 47.9%; *p* < 0.05), with a similar prevalence of nephropathy and neuropathy [36]. In patients with a long duration of type 1 diabetes, higher GADA levels were shown to be more common in patients with peripheral neuropathy and less likely in patients with severe retinopathy [37]. In support of that data, we previously observed in a sample of 461 LADA patients that GADA is associated with peripheral neuropathy; however, that cohort has a significantly longer duration of diabetes compared to this study [38]. There is no consensus, and the majority of cross-sectional studies did not reveal significant associations between specific β-cell autoantibodies and the risk of development of microvascular complications in diabetes [39,40]. It seems that the assessment of diabetes-associated autoantibodies at diagnosis does not help predict the development of future microvascular complications, although higher GADA levels have been associated with subsequent nerve damage [41].

Due to increased LADA prevalence in clinical settings, earlier identification of patients requiring frequent monitoring with the earlier intensification of insulin therapy might be of particular clinical interest. Those with simultaneous GADA, ZnT8, and IA-2 positivity have a more severe form of β-cell autoimmunity, and their residual β-cell function might be preserved by early introduction of insulin therapy [42]. A high GADA titer with ZnT8 and IA-2 positivity in the first year after diagnosis significantly increases the progression toward insulin requirement in patients with LADA [43]. Although a high titer of GADA is generally connected with a shorter insulin-free period, results from the significant and essential United Kingdom Prospective Diabetes Study (UKPDS) suggest that GADA autoantibodies persist for six years after diagnosis of LADA but are not associated with disease progression [44]. Several autoantibodies, more so than high titers of GADA, predict insulin dependence, and the presence of multiple β-cell autoantibodies is highly correlated with a faster decline of islet function in patients with LADA [45,46].

The present study has several potential limitations. First, this was a single hospital-based cross-sectional study with a limited number of study participants that most probably are not representative of the population of LADA. Therefore, the data must be confirmed in prospective studies with more patients. Second, our analyses were based on a single measurement of autoantibodies, which might not reflect the dynamic nature of autoimmunity over time. Third, clinical methods used to diagnose diabetic retinopathy and neuropathy may influence the results, making it difficult to compare findings between studies. Fourth, since our study only included patients from the white European population, there was no racial/ethnic diversity.

In conclusion, our study, which included 189 adult LADA patients, revealed diverse associations between individual-specific β-cell autoantibodies and their combination with clinical and metabolic phenotypes. The patients with a single GADA positivity exhibited characteristics more similar to those with classic type 2 diabetes, while those with positivity to all three autoantibodies had clinical and metabolic characteristics of classic type 1 diabetes. In a clinical setting, it is essential to differentiate these two clinical phenotypes within the adult patient population because the prevalence of LADA nowadays is increasing significantly due to the growing awareness of this subtype of diabetes. A diagnostic approach using multiple specific β-cell antibodies may help identify subpopulations of adult patients with diabetes requiring more frequent monitoring to improve patient outcomes. No association between antibody positivity and microvascular complications of diabetes was found in our study, probably because of the short duration of diabetes and relatively satisfactorily controlled disease. However, the relationship between β-cell autoimmunity and chronic complications of diabetes merits further investigation in long-term prospective studies.

## Figures and Tables

**Table 1:** Clinical characteristics of the GAD-positive adult diabetic patients according to the diabetes-specific autoantibody patterns.

Variable	Entire Cohort	Antibody-Positivity-Related Subgroup				
		GADA Only	ZnT8+GADA	IA-2+GADA	ZnT8+IA-2+GADA	*p*-Value
N	189	91	31	17	50	
% of the total	100	48	16	9	27	
Sex (M/F)	91/98	53/38	11/20	10/7	17/33	**0.0148**
Age (years)	53 (39–63)	56 (42–66)	47 (37–56) *	49 (38–62)	50 (36–60)	**0.0261**
Diabetes duration (years)	4 (2–11)	6 (2–15)	3 (1–7) *	4 (2–8)	2 (1–7) *	**<0.001**
BMI (kg/m^2^)	25.6 (22.0–29.0)	28.0 ± 5.5	24.7 ± 5.6 *	25 (22–27)	24.0 ± 3.7 *	**<0.001**
C-peptide (nmol/L)	0.28 (0.18–0.47)	0.33 (0.22–0.54)	0.3 (0.22–0.43)	0.21 (0.12–0.36)	0.22 (0.15–0.35) *	**0.0038**
HOMA2-B (%)	25.9 (13.4–39.6)	29.4 (15.7–44.4)	18.6 (13.5–33.5)	22.1 (9.4–35.4)	23.9 (9.6–36.7)	0.1228
HOMA2-IRI (1/1)	0.84 (0.51–1.39)	0.98 (0.55–1.71)	0.83 (0.65–1.08)	0.82 (0.32–1.00)	0.64 (0.44–1.20) *	0.0327
FPG (mmol/L)	8.7 (7.0–12.0)	8.5 (6.9–11.3)	9.6 (7.5–13.3)	8.9 (7.2–13.1)	8.5 (6.9–11.5)	0.8432
HbA_1_c (%)	7.4 (6.5–8.7)	7.4 (6.4–8.8)	7.1 (6.6–9.8)	7.1 (6.0–8.3)	7.4 (6.6–8.4)	0.7247
eGFR (mL/min/1.72 m^2^)	97 (85–107)	93 (80–101)	106 (94–118) *	100 (91–109)	100 (87–110)	**0.0011**
U-ACR (mg/mmol)	1.3 (0.6–3.7)	1.5 (0.8–4.9)	1.5 (0.6–4.2)	0.9 (0.63–1.98)	0.8 (0.5–2.4)	0.0548
TSH (mIU/L)	1.80 (1.20–2.77)	1.87 (1.19–2.90)	1.93 (1.20–2.82)	1.52 (1.32–2.05)	1.70 (1.18–2.65)	0.6608

Values are presented as median (interquartile range) or mean ± SD, depending on the data distribution. Chi-square test for categorical variables; Kruskal–Wallis ANOVA for continuous variables. * *p* < 0.05 compared to GADA only subgroup (Dunn’s post-hoc test) (bold values). GADA: glutamic acid decarboxylase antibodies; ZnT8: zinc transporter-8; IA-2: anti-islet-2 antibodies; HOMA2-B: homeostatic model assessment 2 β-cell function; HOMA2-IRI: homeostatic model assessment 2 insulin resistance index; eGFR: estimated glomerular filtration rate; U-ACR: urinary albumin to creatinine ratio.

**Table 2:** Predictors of glucose control (HbA_1_c ≤ 7.0%/N = 100—good control; HbA_1_c > 7.0%/N = 89—poor control) in GADA-positive adult diabetic patients.

Variable	Odds Ratio	95% Conf. Interval	*p*-Value
GADA (U/mL)	1.0008	1.0004–1.0011	<0.0001
HOMA2-B (%)	0.9670	0.9478–0.9865	0.0010
Duration of diabetes (years)	1.0804	1.0200–1.1444	0.0084

Multiple logistic regression analysis. Variables not retained in the model: age, sex, BMI, IRI, IA-2 positivity, ZnT8 positivity. GADA: glutamic acid decarboxylase antibodies; HOMA2-B: homeostatic model assessment 2 β-cell function.

## Data Availability

The data presented in this study are available on request from the corresponding author.
